# Second Report of the Commissioners for Inquiring into the State of Large Towns and Populous Districts

**Published:** 1846-04

**Authors:** 


### Art. II.

1. *Second Report of the Commissioners for inquiring into the State of Large Towns and Populous Districts.* 2 vols. 8vo.

2. *A Bill for the Improvement of the Sewerage and Drainage of Towns and Populous Districts, and for making Provision for an ample Supply of Water, and for otherwise promoting the Health and Convenience of the Inhabitants.*

I. Report of the Commissioners. In this, their second report, the commissioners observe, that until the publication of the reports made to the Poor Law commissioners in 1839 upon the condition of the poorer classes of her Majesty's subjects in certain parts of the metropolis, followed by the report of a Select Committee of the House of Commons, in the year 1840, "on the Health of Large Towns and Populous Districts," the extensive injury to the public health, now proved to arise from causes capable of removal, appears to have escaped general observation, while the means of remedying the evils by improvements in drainage, or by other structural arrangements, as have been carried into operation, have been executed more with a view to the appearance of the town or the comfort of a portion of the inhabitants, than directed to maintain the health of the whole community. They also state that subsequent investigations and reports have excited increased attention to the importance of providing for the physical condition of the poorer inhabitants of large towns. The wealthy and intelligent classes resident in them are now for the most part becoming alive to this great question, and to the necessity of providing for the removal of those causes which tend to vitiate the air in the quarters occupied by the poor, and especially in those most densely crowded.

There can be no doubt that the course of inquiry the commissioners adopted for obtaining information was eminently judicious, and has mainly led to these results. By calling to their aid the assistance of the most influential and intelligent of the inhabitants, through whose means and local knowledge the peculiar conditions of many localities were closely investigated, scenes of misery and neglect were exposed to their view, of which many were previously ignorant, and their attention directed to causes of disease, arising from the defective state or absence of proper structural arrangements.

The necessity and utility of sanitary arrangements for towns is by no means a new topic of discussion within the profession. Even the idea of making the refuse of towns serviceable to agriculture, and a source of wealth to the country (developed by Mr. Smith, of Deanstown, in his report on York and other towns), was broached so early as 1798. In the second number of the 'American Medical Repository' of that year, Dr. Mitchell proposed, "to convert the mass of nuisance, which we now are with such happy success engaged in removing from the city of New York, by the powers of vegetation, from poison to wholesome articles of food." His plan was, that lime, potass, and soda, should be thrown about the streets to neutralise the emanations, and make the refuse more efficacious as manure. In 1802, the question of town drainage was again mooted in the United States and England. In that year, Dr. Patterson, of Londonderry, thus writes to the editor of the 'London Medical and Physical Journal:' "Perhaps some of the readers of the Medical Journal may be able to give an instructive answer to the following question, asked by Dr. Miller, of New York: 'Can you direct me to any source of full and satisfactory information concerning the best method of discharging from cities the filth of animal and vegetable kinds constantly accumulating in them, and especially the filth of privies? We are endeavouring to make some improvements of this description in New York, and wish to be instructed and assisted by the wisdom and experience of older cities.' Give me leave, on this occasion," adds Dr. Patterson, in the language of the day, "to second this wish of Dr. Miller, and to interest on this occasion your ingenious and philanthropic correspondents." Either the editors had no correspondents of the required description, or more probably their ingenuity and philanthropy were not equal to the emergency, for Dr. Patterson's appeal met with no response.

We believe that, although there was a general impression on the minds of professional men as to the noxiousness of emanations from putrid or decaying animal or vegetable matter, less specific information was possessed by them, previously to the appearance of Mr. Chadwick's famous report, than the majority would willingly allow. Stinks on a small scale were unnoticed; they crept through the net of professional criticism. Stinks on a large scale were adjudged offenders, but were too great to grapple with, and were passed over with a shake of the head and a solemn protest. Formerly the judgment of the public was not enlightened; consequently the appeals of the profession to the dull and ignorant authorities might as well have been made to a statue of Memnon. It is by the active collection and diligent diffusion of information on matters relating to the public health, that the Health of Towns' Commission have done such good and lasting service to the public.

As the subjects specified in her Majesty's commission were essentially of a practical character, the commissioners have endeavoured to avoid as far as possible the discussion of the theoretical causes of disease. All the medical witnesses examined before them are unanimous as to the injurious effects produced by emanations from animal or vegetable matter in a state of decay, whether they act as direct or contingent causes of disease; and they are quite concurrent in their opinion that the existence of such causes and their prevalence have been sufficiently ascertained to require the interference of the legislature. The presence of such emanations, whether they be derived from stagnant ditches, from open cesspools, or from accumulations of decaying refuse, is a great cause of disease and death, not confined to the immediate district in which they occur, but extending their influence to neighbouring and even distant places.

It is too commonly supposed, the commissioners observe, that the evils above adverted to are the inseparable concomitants of poverty; and doubtless, so long as the inhabitants of the most neglected and filthy abodes in crowded cities are unable to provide for themselves better and healthier dwellings, sufficient light and air, more open situations, effective cleansing and drainage, and adequate supplies of water, their vigour and health are undermined, and their lives shortened by the deleterious external influence consequent upon the want of efficient arrangements for securing the above objects.

The commissioners farther advance, as a result of their inquiries, that other diseases besides fevers, namely, scrofula and consumption, are developed by injurious emanations and impure air; that the ascertained excess of deaths amongst artisans is in a great degree due to the defective ventilation of their places of labour; that the crowded and impure courts are distinguished by a high infantile mortality, and an increased proportion of births; and that many of the pecuniary embarrassments and much of the wretchedness and moral degradation of the poor may be traced to the physical degradation consequent on the neglect of hygienic measures. One of the facts which came to their knowledge, although not within the terms of their commission, they express themselves as bound to report on; it is the "terrible practice," as they term it, of giving opiates to children; terrible because it induces not only death in its victims, but mental debility and imbecility in those who survive the administration of the narcotic.

The following is a short outline of the measures they propose to be adopted: their main reasons for these will follow.

"We are of opinion that, for the effectual correction of the evils above adverted to, additional legislative measures are requisite. It is necessary that the Crown should have power to inspect and supervise the execution of all general measures for the sanatory regulation of large towns and populous districts.

"That the local authorities entrusted with the execution of such measures should be armed with additional powers, and that the districts placed under their jurisdiction should in many cases be enlarged, and made coextensive with the natural areas for drainage.

"We recommend that the necessary arrangement for drainage, paving, cleansing, and an ample supply of water (the most important matters conducive to health) should be placed under one administrative body.

"We also urge the necessity of some general sanatory regulations relative to buildings and the width of streets, and that low lodging houses should be placed under public inspection and control." (p. 11.)

It is a remarkable circumstance that most serious attention was given to works of drainage from the earliest periods of our constitutional history. The earliest fundamental provisions have been based upon the footing that such works, as well as measures for the maintenance of the free flow of running waters, were of general public and national, rather than of exclusively local, consideration. It is held, by the first legal authorities, to be one of the prerogatives of the Crown to issue commissions for the protection of the population, by the enforcement of proper works of drainage, and this prerogative appears to have been exercised by the issue of special commissions, as well after as before the passing of statutory provisions on the subject. The intervention of the Crown was often urgently sought for the public protection against the injurious encroachments of private interests upon the great public watercourses for mill power or for fishing-weirs. The 16th chapter of Magna Charta is a defence of the public rights against the growth of such encroachments. The fourth statute of the 25 Edw. III, c. 4, provides for the putting down of mills, weirs, dams, and other obstructions, and commissions appear to have been issued from time to time to see to the execution of the laws provided thereon. The commissioners give numerous examples of royal commissions for these purposes, and note the various modifications they have undergone from time to time until absorbed in modern legislation.

The inefficiency of modern, and especially local acts, seems to be partly due to the uncertain tenure by which the authorities hold office, partly to local influence, and partly to inefficiency of the powers given by law. It appears that ignorance of the authorities, (usually tradesmen, merchants, or lawyers,) has also been no trifling impediment to improvement.

The first and most important step, the commissioners observe, in providing for the efficient and economical execution of any plan of drainage, is the preparation of an accurate general survey, upon a large scale, of the area which it is proposed to drain. This view is supported by a large mass of valuable and important testimony, proving it to be the necessary preliminary to any such work. The extent of country to be comprised within the jurisdiction of any local authority should be the entire natural area for drainage.

At present no such plans or surveys are accessible to builders or others engaged in works requiring a knowledge of the level of the adjacent lands. Hence serious losses have been entailed on the public by the construction of sewers and drains at improper levels, and of a capacity insufficient for the probable wants of a future population; and houses have been placed in situations, regardless of the means of drainage. Great loss and inconvenience from this cause have very generally occurred, and even very lately it has become necessary to enlarge and deepen some of the sewers recently put in.

The prevailing want of information among the surveyors and other officers having the charge of the drainage of towns, regarding the levels of the sewers, and frequently even the entire ignorance of their existence, may be traced to the absence of any proper survey. At Bristol, the first attempt to form a complete map of the sewers was commenced during the inquiry of the visiting commissioners, and in the town of Preston it was a work of several weeks to open the streets in order to ascertain the lines and depths of the sewers. In some large towns, as Wigan, Rochdale, and Bolton, there is not the slightest knowledge of the plans of the sewers. The charge by the Ordnance Office for surveying such a parish as St. Clement Danes, and laying down contour lines, with the sewer, gas, and water pipes, would not amount to more than 8*s*. per acre; but if such additions were made while a survey for the Ordnance map was in progress, the extra cost would be reduced to 1*s*. 4*d*. per acre, or to 6*d*. only, if the levels merely are taken and bench marks inserted; the scale in that instance would be 60 inches to the mile, but it appears that the extent of the scale would scarcely make any appreciable difference in the cost. This cost would vary in a trifling degree, according to the density of the dwellings and population in a given area, but it has been calculated that for laying down the levels, an outlay of from 30*l*. to 40*l*. would be enough for the average size of towns containing 20,000 persons; from 100*l*. to 120*l*. for towns of 60,000; and from 200*l*. to 250*l*. for those of 120,000 inhabitants.

The commissioners in the course of their investigations noted numerous evils to arise from limited jurisdiction for drainage. The suburban houses of many large towns are under no jurisdiction whatever, and sewers and drains are constructed or not, according to the whim of builders or occupiers. The outfalls of the natural drainage in several instances is occupied by dams, &c., for obtaining mill-power, as at Bradford, Halifax, and other places. The upper and lower parts of a town again are under a different set of authorities, and these clash and obstruct each other. The commissioners think the Crown should be empowered to define and to enlarge, from time to time, the area for drainage included within the jurisdiction of the local administrative body.

The necessary works for the drainage and sewerage of towns demand a surveyor of competent information. It appears, however, that local improvement acts do not require any qualifications, nor do they advert to the necessity of skill and experience in the officer; indeed, many of them do not so much as allude to the office. Everybody knows that merit does not always secure the favour of public bodies; so the blind leads the blind, and sad botches they make. The eminent truth of the following remarks is obvious.

"All local works of improvement should be planned, and their execution superintended by a person having a competent knowledge of engineering. New subjects, connected most closely with the general health of the community, are now constantly attracting the attention of persons engaged in works of construction. The importance of the questions of ventilation and warming having been fully established by recent investigations, particularly demands the attention of the architect and engineer. The important duties which may in future devolve upon the officers charged with the construction of works, and the large discretionary power that must be vested in them, will undoubtedly render it necessary to establish some mode of testing the competency of persons offering themselves as candidates to fill such situations. Some assurance should be given to the public that the persons intrusted with these responsible duties are properly qualified." (p. 36.)

The deduction made from inquiries on this point is embodied in the following:

"We, therefore, recommend that the local administrative body appoint the executive and other officers under it; that the appointment and dismissal of the chief surveyor be subject to approval; that such officer produce proof of his qualification for the office to which he shall be appointed, and, if required, be subject to an examination." (p. 37.)

Hitherto public bodies have been found slow to act for the general good, and easily moved by private feelings in the removal of nuisances, and the regulation of matters involving the public health. The commissioners observe, that the contentions of parties and the influences of local interests frequently impose a serious obstacle to any scheme of improvement, from the unfounded fear that their interests will be affected. Such persons frequently obtain great influence in the decision of questions in relation to any alterations calculated to effect improvement in the condition of the working classes. It seems a fair proposition, that where the public welfare requires it, the inhabitants generally should be made responsible for the execution of the duties imposed upon them by law, for sanatory objects, on the same principle as they are now liable, for the public benefit, to repair the highways. On these and other grounds, the commissioners

"Recommend that, upon representation being made by the municipal or other authority, or by a certain number of the inhabitants of any town or district, or part thereof, setting forth defects in the condition of such place, as to drainage, sewerage, paving, cleansing, or other sanatory matters, the Crown direct a competent person to inspect and report upon the state of the defects, and, if satisfied of the necessity, have power to enforce upon the local administrative body the due execution of the law." (p. 39.)

The enormous defects in the construction of main and branch sewers in many towns, and their total want in many courts, alleys, and close thoroughfares, have been strongly pressed upon the attention of the commissioners. The result of their deliberation on this point is, that the construction of sewers, branch sewers, and house drains, ought to be intrusted to the local administrative body.

After one or two recommendations as to the modes in which the necessary funds should be raised, the commissioners proceed to the important matter of paving. They do not seem, however, to have devoted much, if any, attention to the comparative merits and hygienic relations of the various kind of pavement lately brought into notice, but simply content themselves with expressing the opinion, that the whole of the paving, and the construction of the surface of all streets, courts and alleys, be placed under the management of the same authority as the drainage, and that the limits of jurisdiction for both purposes, wherever practicable, be co-extensive: also that the principle before submitted in respect to the cost of making drains and sewers, and the equitable distribution of the expense, be adhered to in the case of laying out, levelling, and paving of streets, courts, and alleys; but for the purpose of insuring the greatest efficiency and economy in the execution of the work, they recommend that it be performed by the local public officers.

Few of our readers are unacquainted with the distressingly filthy condition of the streets and courts occupied by the labouring classes of towns; of the numerous and formidable diseases arising therefrom, and of the difficulties such a condition raises against the successful treatment of disease. All this is *proved* by the most extensive and elaborate statistical data in the reports of the commission. It is a more interesting question *now* to determine the mode in which the refuse of a town can be best removed.

The earliest regulation appears to have been this; that all householders should cleanse that portion of the street before their respective houses, and to heap up the dirt and soil in preparation for the scavengers. Wc find such duties prescribed in a very early act for Liverpool (21 Geo. II, c. 24). It is thereby enacted, that the occupiers shall sweep their portion of the streets at least twice a-week, on every Monday and Thursday, or oftener if required, and that the scavengers shall attend every Tuesday and Friday, "or oftener if occasion be." It appears, however, that although the provisions of this act, positively directing the scavengers to cleanse all the streets twice a-week, were in force up to the year 1842, it was the practice to cleanse the minor streets only once a-week, and the others, where there was less traffic, when required.

The manifest inconveniences of such a system naturally led to the appointment of scavengers paid by the householders. In Aberdeen, the local act requires the appointed scavengers to cleanse the foot-pavements, and the whole of the streets, closes, courts, &c. every day, under a penalty. This work is done at a profit of £600 a-year to the city, and in other towns in Scotland similar examples are found. The commissioners observe that the law which the legislature has made so stringent at Aberdeen, and which has been carried into execution there and at Edinburgh, may with equal advantage be applied generally to all parts of Great Britain.

The removal of dirt, ashes, and rubbish from all houses and premises is another important point in town-cleansing. This has also been made a profitable affair. In the metropolis the ashes are an article of considerable trade. The contractors are in the habit of paying large sums of money to the parishes for the right to collect them, and the large parishes frequently make a considerable profit from the exercise of this right, which aids in the diminution of the rates, after paying the expense of cleansing (in the mode considered sufficient by them) all the streets and roads in the parish. The commissioners, for these and other reasons, recommend that the provisions in local acts, vesting the right to all the dust, ashes, and street refuse in the local administrative body, be made general; and that the cleansing of all privies and cesspools at proper times, and on due notice, be exclusively intrusted to it.

The removal of nuisances is the next question considered by the commissioners. The first noticed is that of "midden-steads," or dung heaps. Perhaps there are no more general and frequent sources of fever than these. In Ireland they probably cause the greater proportion of fever cases. That country, in fact, may be medico-topographically described as the land of dung-hills and fevers. But in England, with all its pretensions to cleanliness, towns are bad enough. Let us take Sunderland as an example. In this borough, on the testimony of its inhabitants, there are no fewer than 182 public midden-steads, receptacles for filth of all kinds, which are stated to constitute one of the greatest nuisances within the borough. They are generally situated in the close, narrow streets and lanes inhabited by the poorer classes, and are frequently resorted to by them. In some cases these midden-steads are actually in the basement floor of a dwelling-house, the upper stories of which are occupied as bed-rooms, &c. The contents of these midden-steads are afterwards conveyed to large depôts, of which there are two in the parish, one very lately advertised as containing 1000 tons for sale. This belonged to the borough.

The state of the slaughter-houses are also an almost constant source of complaint. They are very rarely placed under any regulations with regard to the constant removal of the animal refuse, their proper ventillation, or a sufficient supply of water to ensure due cleanliness. The improper situations in which these places are found, sometimes even under dwelling-houses, and the effect produced upon the health of the inhabitants, is described in the report on the towns of Lancashire. In these towns slaughter-houses are found below dwelling-houses, the smell in which was most insufferable. In many of these cases the inhabitants looked pale and sickly, and diarrhœa frequently prevailed, although absent from the court, contiguous. Yet the state of the law prevents any interference with the manner in which these slaughter-houses are conducted. True it is that aggrieved parties may indict the occupiers of the premises, but they being labouring men, can neither afford the time nor money to pursue such indictments, nor do they belong to a class aware of the pernicious effects arising from the presence of decomposing refuse. The uncertainty of the result, moreover, is alone sufficient to deter even those who have both the means and the inclination to suppress them. In scarcely one instance, however, in which shambles or slaughter-houses have come under the observation of the commissioners, either in the metropolis or in the provincial cities or towns, have there been found in force any regulations or authoritative supervision to compel the speedy and regular removal of offal from, or the efficient cleansing of such places. They have, on the contrary, been found to be, almost without exception, centres for the diffusion of noisome influences, affecting, with more or less intensity, the immediate vicinity, deteriorating the sanatory condition of the surrounding population, commonly poor and dense, as recorded in the local reports of the commissioners, and in a more remote degree vitiating the general atmosphere of the town, and thus becoming a nuisance to the inhabitants at large.

A second evil and nuisance, necessarily contingent upon the locality of slaughter-houses, however stringently supervised and regulated, in the midst of large and populous towns, is the quantity of animal ordure deposited upon the public streets and thoroughfares leading to such slaughter-houses, which, besides forming a most offensive addition to the ordinary surface-filth, excites and accelerates its decomposition. This evil is augmented in the ratio of the size of the town, and where, as in London, most of the surface-filth of the streets is washed down into the sewers, the continual passage of cattle, sheep, and pigs, in the neighbourhood of the intra-mural slaughter-houses, must materially increase the amount of that decomposing matter, the emanations of which are constantly escaping from the untrapped gulley-holes, to infect the atmosphere of the metropolis. Nor ought the occasionally fatal injuries, and the constant peril of life and limb, incurred by the inhabitants of large towns, the streets of which are so frequently traversed by goaded and over-driven cattle to be overlooked.

The practice of keeping pigs is a most odious nuisance. They are eminently stinking animals when in a state of confinement. In Birmingham there are 1600 pigsties: at Sunderland pigsties were said to be the *foci* of cholera. It is remarkable that, although there are by-laws in several corporate towns forbidding the accumulation of offensive matter occasioned by these sties, and by slaughter-houses, they are totally disregarded, as, for example, Newcastle-on-Tyne, Sunderland, Norwich, &c. The powers of courts-leet have been equally inefficient. This inefficiency manifestly arises from the reluctance of a party to bear witness against his neighbour. On this point of health-police, the commissioners observe :—

"By the introduction of better regulations on the subjects which we have now been noticing, it is to be hoped that the occasions for any interference of this kind will become less frequent; but as the most constant attention is required for the punctual enforcement of any laws or regulations, we are of opinion that an officer should be appointed in each town, who, in addition to other duties that may be placed under his charge, should be required to report upon any neglect on the part of the scavengers, or any infringement of rules for the prevention of nuisances, or of any other matter affecting the health of the inhabitants, and, if necessary, to commence proceedings in his own name, and as an informer on the part of the public, for the punishment of offenders before the magistrates Such an officer would receive much valuable assistance in the execution of his duties, and the public would be checked in their infringement of the law, if the police were directed to report upon any breach or neglect of it. These public servants, now generally a numerous and efficient body in each large town, although the constant witnesses of such offences, are not charged with the duty of reporting them to their superiors, or any officer empowered to correct them. It has been represented to us that this duty could be most efficiently and conveniently executed by the police, without any serious addition to their labours, or increase of expense to the inhabitants." (p. 79.)

The commissioners propose that the keeping of pigs, the collection of dung, and the establishment of slaughter-houses, within the precincts of towns, should be absolutely forbidden, and summarily abated. Perhaps, as regards the keeping of pigs, it would be as well to make private interest subservient to public duty, and adopt such arrangements as will encourage the sale of pig-wash and offal-food to wholesale pig-feeders.

We think it would have been as well had the commissioners directed their attention to the condition of stables and mistals, or cow-houses, in large towns. The confinement of large numbers of cows in a limited space, and surrounded by the impure atmosphere of a large city, cannot but be injurious to the health of the animals, and render their milk unwholesome. There are reasons for suspecting that the use of such milk may induce phthisis. The extension of railway communication will now render the prohibition of cow-keeping in large towns feasible, as the milk may be brought from the country in a few minutes.

The abatement of the nuisances arising from the smoke and noxious emanations from mills and chemical manufactories, is proposed to be effected by requiring the combustion of smoke, arising both from mills and steamers, and such other appliances as may be requisite. The same power, the commissioners observe, that advances the chemistry of the arts and manufactures has multiplied the means of controlling and destroying offensive and injurious products from chemical operations. Taking, therefore, into consideration the facilities that now exist for preventing such noxious emanations, we feel convinced that much advantage will accrue both to the manufactory and to those who dwell in its vicinity by the right application and more careful investigation of the means which have already been put into successful operation for abating similar evils. The recommendation on this point is, that in cases where complaints shall be substantiated that the inhabitants of any house, street, or district, in towns, are injuriously affected by the noxious exhalations of any factory, power be given to the local administrative body to ascertain the cause of such exhalations, and to take legal proceedings for the abatement of the evils, in the event of such evils not being removed on due representation.

The necessity of an ample supply of water to towns, as the basis of efficient sanatory measures, is dwelt upon at considerable length by the commissioners. They observe, that the filthy condition which the abodes of the poorer classes so constantly exhibit, has produced a very general impression that they are not capable of appreciating the advantages and comfort either of personal or domestic cleanliness. The information derived from the investigations of the commissioners, and the evidence obtained through other channels, has convinced them that this is a most erroneous view of the feelings and wants of those persons; and they are most desirous to correct this impression, which, if it were well founded, would form a barrier to any prospect of improvement, and would render nugatory the recommendations that they may subsequently make for facilitating increased supplies of water. The general habits of the poor, with regard to cleanliness, must not be compared with a high standard; their daily occupations, and the nature of their employments, are such as frequently render personal cleanliness comparatively unattainable, and unless every possible facility is afforded for this end, they soon become insensible to its importance. The present difficulty, and the labour, after a hard day's work, of obtaining water, has a very great effect on their economy, their habits, and their health. The obstacles to the maintenance of domestic or personal cleanliness soon produce habits of personal uncleanliness, which rapidly lower both the moral and physical condition of a whole population. Wherever, on the contrary, a better supply has been afforded, the poor have appreciated the boon by readily paying for it, and using it, to the manifest improvement of their health and comfort. In addition to the necessity of an ample supply for domestic use, the watering and cleansing of the streets and drains, and the ready extinction of fires are dependent upon civic hydraulics.

Many towns are most imperfectly supplied with water; but there is great difference in this respect. At Longton, a town in the Potteries, of 2000 houses, nearly all have a separate and constant supply. Bath is also eminent for its ample provision. The system of common stand-pipes for the supply of the poorer classes is strongly objected to by the commissioners. It is obvious that they must watch their opportunity of collecting water during the period that it is turned on, and those who are engaged in occupations from home necessarily lose their chance of getting a supply. This inconvenience is particularly felt in districts where women and children have much employment. When pipes are not laid on to each house, much labour is expended in fetching the water, and time is lost in waiting for their turns to fill their vessels. Where many persons are collected, as frequently happens, quarrelling naturally ensues for precedence, while serious injury is often inflicted upon the morals of the better portions of the population. The advantages of a system of constant supply are so clear, and have become so generally known since the inquiries of the commissioners have been made, that we believe in a few years it will be generally adopted.

In estimating the quantity of water to be supplied, the commissioners think that in all cases where an ample supply can be prôcured, it ought not to be calculated at a less rate than twelve gallons per diem for each individual of the population. The quantity required for public purposes will vary according to the situations and other peculiarities of towns. The water necessary for flushing the sewers will diminish as the natural advantages for drainage are greater; and the quantity used for watering the streets will vary according to the materials of which they are constructed. A more abundant supply may lead to the adoption of a system of washing the dirt from the foot-pavements, and other roads which are constructed of such materials as will admit of this mode of cleansing. The recommendation of the commissioners is, that it be rendered imperative on the local administrative body to procure a sufficient supply of water for these purposes. They strongly object to the competition of rival companies, because it eventually fails to attain the object for which it is begun. They rather recommend that where any independent body has the management of the supply of water, it be liable to comply with the demand of the local administrative body on equitable terms; that the local administrative body be empowered to purchase the interest in water-works, subject to the control of the Crown, whenever the proprietors are willing to dispose of them: that on the establishment of new companies it may be made a condition that the local administrative body be enabled to purchase the works after the lapse of a certain number of years, upon certain terms, and upon a rate of interest to be fixed; and that, with a view to economy, competition between water companies be discouraged as far as practicable. That the supply may be universal, and therefore afforded at an easy charge to each individual, the commissioners recommend that as soon as pipes are laid down, and a supply of water can be afforded to the inhabitants, all dwelling-houses capable of benefiting by such supply be rated in the same way as for sewerage and other local purposes; and the owners of small tenements be made liable to pay the rates for water, as they have recommended in respect to drainage.

The system of public baths and laundries, begun at Liverpool, has extended to London and other towns; another recommendation of the commissioners is, that every facility be afforded to furnish ample supplies of water to public baths and wash-houses that may be established for the use of the poorer classes.

The extinction of fires they propose to secure by a system of fire-plugs opening into mains filled with water at high pressure. As we have alluded to this point in our notice of the first report, we need not say more.

The regulations for buildings constitute an important part of health-police. It appears that the legislature has hitherto sanctioned but few local acts containing provisions for regulating the disposition of land as regards the width of streets, and the space to be allotted for houses; and restrictions are rarely placed upon the mode of constructing houses, either with a view to prevent the extension of fire, or to provide the occupants with those comforts and conveniences which are now considered necessary parts of every dwelling. The results of this neglect may be seen in the narrow courts, cellar-dwellings, and alleys, abounding in all our large towns, but pre-eminently so in Liverpool, Manchester, Preston, and Birmingham. To abate the existing evil, the commissioners recommend that, subject to proper control, the local administrative body be empowered to raise money for the purchase of property for the purpose of opening thoroughfares, and widening streets, courts, and alleys, so as to improve the ventilation of the densely-crowded districts of towns, as well as to increase the general convenience of traffic. With a view of insuring better external ventilation in future erections, they recommend that courts and alleys be not built of a less width than twenty feet, and that they have an opening of not less than ten feet from the ground upwards at each end; the width of the court being in proportion to the height of the houses. With respect to cellars, they recommend that after a limited period the use of them as dwellings be prohibited, unless the rooms are of certain dimensions, are provided with a fireplace, a window of sufficient size and made to open, have an open space in front, and that the foundations be properly drained.

The sad neglect of builders of cottage tenements in not supplying them with suitable privies, is strongly commented on by the commissioners, as inflicting extensive injury on the health, decency, and morals of the poor. The large number resorting to these places deprive them of all privacy. To save the space occupied by a privy in each house, a number of them, for the use of an entire population of a court, are commonly crowded together in one corner, and not unfrequently placed under other dwellinghouses. The owners of the property are themselves also sufferers. Many instances occur where the walls of the adjoining houses are constantly wet with fœtid fluid, which frequently affects the atmosphere of the rooms so as to render it impossible to keep food for one single night without its becoming tainted. The walls of the houses receive considerable damage, and the foundations are completely saturated with the foul water that percolates through from the cesspools. The deterioration of property from this cause is very considerable. Added to this, a constant loss is incurred by the inability of tenants to pay their rents, from sickness, and not unfrequently from the impossibility of finding persons reduced so low in the scale of society as to occupy such abodes. The commissioners recommend that the local authorities should have a discretionary power of compelling the erection of privies where circumstances permit, and that all new houses be provided with them.

Fires are rare in the dwellings of the poor. Indeed, the tendency of the evidence they have collected on this point has led the commissioners to the conclusion that any general interference with the minute details of buildings for the poorer classes in the great majority of towns in England and Wales is unnecessary. With regard to ventilation, their views are very different. It appears that architects and builders rarely make any provision in buildings constructed by them for a regular supply of fresh, or the removal of vitiated air, beyond what is afforded by the windows, door, and open chimneys. All practitioners know how perseveringly the poor (and the rich too) close every crevice that will admit cold air. Notwithstanding the apparent difficulties with which the ventilation of private dwellings is surrounded, a minute examination of the circumstances of the case has convinced the commissioners that no field of improvement holds out a more promising result than that which may be anticipated in future from the more successful ventilation, even of the humblest dwellings. But although the propriety of enforcing the introduction of a system of ventilation has been urged upon the commissioners, the general balance of opinion is adverse to that view. In this conclusion they concur; and although attaching the utmost importance to the introduction of some means of purifying the air in the abodes of the poor, they cannot recommend the adoption of compulsory provisions for this purpose, which, even if capable of enforcement, must lead to a most objectionable interference with the privacy of domestic life. The application of proper principles must be the result of a more general acquaintance with the subject on the part of individuals. Places of public assemblage and resort, especially schools, ought however to be under supervision. They recommend also that when, on the complaint of the parish medical or other authorized officer, any house or premises are in such a filthy and unwholesome state as to endanger the health of the public, and an infectious disease exists therein, the local administrative body have power to require the landlord to cleanse it properly, without delay; and in case of his neglect or inability, to do so by its own officers, and recover the expense from the landlord.

Medical practitioners conversant with the domestic condition of the poor must be well acquainted with the miserable state of the common lodging-houses. Their crowded condition is really awful. Instances occur frequently where the beds are placed in tiers one above the other. Dr. Howard, who has had great experience from his connexion with the fever-wards in the hospital at Manchester, states that he considers the lodging-houses as the most frequent source of infectious fevers in Manchester, and he ascribes the permanence of the infection to the want of cleanliness in the beds, which are rarely purified, even after having been occupied by patients suffering from fever. In Scotland, examples have come under the notice of the commissioners in which the local authorities have power to license all the lodging-houses, and to issue regulations for their proper management. No such regulations have been adopted, it appears, in England and Wales, but they have been found to operate very successfully in Calton, which forms part of the borough of Glasgow. There the commissioners of police limit the number of persons to be accommodated in each house licensed; they require that the house shall be whitewashed periodically, and that in other respects due attention shall be paid to cleanliness; and above all, that immediate notice shall be given of the occurrence of any case of sickness. Under these regulations, many ill-conducted lodging-houses, the common resort of the infamous of both sexes, have been suppressed; while those now remaining, being licensed, are under the more direct control of the commissioners. Similar measures are recommended for adoption in England.

The appointment of a medical officer of inquiry is next adverted to by the commissioners. We subjoin this part of their report at length:

"The most eminent medical witnesses concur in declaring, that it is by the careful observation of the causes of disease and mortality operating upon large classes of the community, that the mode and extent of their operation may be ascertained, and the power of diminishing and preventing them be acquired. For this purpose the appointment of an officer, whose duty it would be to direct his undivided attention to such causes, would in our opinion be a public benefit, more especially to the poorer classes, and might be advantageously employed in making investigations into matters affecting the sanatory condition of the district under his charge. We therefore recommend that the local administrative body have power to appoint, subject to the approval of the Crown, a medical officer properly qualified to inspect and report periodically upon the sanatory condition of the town or district, to ascertain the true causes of disease and death, more especially of epidemics, increasing the rates of mortality, and the circumstances which originate and maintain such diseases, and injuriously affect the public health of such town or populous district." (p. 122.)

This part of the report concludes by recommending the establishment of public walks, and calling attention to the evils of intra-mural sepulture.

The drainage of the metropolis evidently occupied the particular attention of the commissioners, and a lengthened exposition of the early and existing laws regarding it is given, and will interest the metropolitan practitioner, to whom we particularly recommend its perusal. It is illustrated by two large and valuable coloured maps, the one showing the drainage and the other the water supply of London.

We subjoin the general conclusions of the commissioners, as containing most valuable truths:

"In submitting to your Majesty the measures we recommend for ameliorating the physical condition of the population inhabiting large towns and populous districts by improvements in drainage, cleansing, ventilation, and the supply of water, we must again express our deep conviction of the extent, importance, and difficulty of the subject—a conviction strengthened by the continuance of our investigations. The most important evils affecting the public health throughout England and Wales are characterized by little variety, and it is only in the degree of their intensity that the towns exhibit the worst examples of such evils. Villages and clusters of houses inhabited by the poor are often under the influence of the same causes of disease, though their effect in such situations may be frequently rendered comparatively slight from the more free circulation of the external air. The vitiation of the atmosphere from over-crowding, and the absence of proper ventilation in individual apartments, produces in the rural districts the same disease that arises from the same causes in a town population.

"Though we venture to consider that the recommendations we now lay before your Majesty will, if sanctioned by the legislature, tend to diminish the evils into which it has been our duty to inquire, we cannot conceal from ourselves that in many cases a considerable time must elapse before permanent structural arrangements can be placed on that footing which their importance requires. Though those, who may be specially intrusted with the execution of the legislative powers recommended, will be enabled, by an earnest discharge of their duties, to accomplish great good, we still look to the co-operation of the public for important aid in the removal of those causes of disease to which the poorer classes of your Majesty's subjects are more peculiarly exposed; we do this the more confidently from the interest that has been recently manifested so generally on this important subject, and from the extent to which causes affecting public health have been made known through so many different channels, leading to the introduction of simple, economical, and highly beneficial improvements even in the humblest dwellings. With such co-operation, we have the greatest confidence that vast physical benefits will ensue, and that they will be accompanied by a corresponding improvement in the moral and social condition of the poorer inhabitants of large towns and populous districts." (p. 137.)

II. The Health of Towns' Bill. Before noticing the appendix to this report, we will draw the attention of our readers to the Health of Towns' Bill, brought in at the close of last session by Sir James Graham and the Earl of Lincoln. We are induced to adopt this as the more natural arrangement of our subject, because the enactments of the bill are mainly founded upon the recommendations of the commission. As it is a bulky document, extending to 118 folio pages, an abstract will, we are sure, be acceptable to our readers.

Clause 1. Limits the operation of the act to England and Wales, exclusive of the metropolis. Clause 2. Enacts that it shall be lawful for one of her Majesty's principal secretaries of state to nominate and appoint a sufficient number of fit and proper persons, of competent skill and science, not exceeding ———, to be inspectors, for the purpose of superintending and otherwise assisting in carrying the act into execution, and for performing the other duties required thereby. Inspectors are to inquire into and report on the state and condition of any town or district, particularly in respect to the state of the drainage thereof the quality, and quantity of water supplied to the inhabitants, the average amount of mortality among the population, and generally on the sanatory condition of such town or district, and on any other matters or things which may be deemed necessary and proper, for the purpose of enabling her Majesty to judge of the necessity and expediency of ordering the provisions of this act to be in force within any such town or district. They are also to define the boundaries of towns and districts, for the purposes of the act; to divide them into wards, apportion the number of commissioners, &c. The Crown may make an order for enforcing the act. Every order in council to be published in the London Gazette.

Clauses 14 to 30 regulate the election of commissioners by the rate-payers; 31 to 41 regulate the selection of commissioners from town corporations, justices of the peace, and local boards of trustees. Clauses 42 to 72 regulate the qualifications and proceedings of the commissioners. Clause 73 provides for the appointment, subject to approval by one of her Majesty's principal secretaries of state, and with such salary as shall be approved of by him, to be paid by the commissioners, a person duly qualified as a civil engineer, to act as a local surveyor of the drainage and other works authorized under the provisions of the act. The surveyor cannot be dismissed without the sanction of the secretary of state. Clauses 74 to 84 provide for the appointment of a clerk, treasurer, and collector, and regulate their proceedings. Clauses 84 to 89 refer to the accounts and to the duties of the auditors, who are appointed by the Crown. 90 to 96 empower inspectors to visit towns and make reports; to attend the meetings of the commissioners; when called upon by the latter, through the secretary of state, "to prepare plans of any new works, additions, alterations, or amendments, that may be required for the drainage of the houses, streets, courts, open spaces, and roads, within any such town or district, including provisions for properly trapped drains or channels of conveyance for the removal of all waste water and refuse from the houses, and from the surface of such streets and places as aforesaid; and also to draw on such plan the most advantageous lines for main-sewers, and the best outfalls for clearing the whole area or district of surplus moisture, and for effecting the drainage of the subsoil; and also to point out the best arrangements that can be made for obtaining supplies of water, and report thereon in manner hereinafter provided; and also, if required, to examine and point out the most appropriate means and sites for the collection and sale of such filth and refuse, and its application as manure for agricultural purposes." The inspector must also prepare estimates for such proposed works, and advise the commissioners generally. Clause 96 provides that the report of every inspector shall be forwarded to one of her Majesty's principal secretaries of state, who shall take the same into his consideration; and if it shall appear to such secretary of state that the provisions and regulations of this Act have not been complied with, or that any of the commissioners have exceeded or contravened the powers and provisions of this act, then such secretary of state shall certify the same to the attorney-general, who shall proceed to enforce the law against such commissioners. This is an important part of the bill. Clauses 97 to 99 provide for the construction of maps of the several districts. 100 to 109 repeal or amend existing acts, and provide for compensations. Clauses 111 to 124 regulate the paving. 125 to 140 prescribe the management of drains, public and private sewers, and of cleansing. No houses are to be built without drains; vaults and cellars are not to be made under the street, without consent of the commissioners; gully holes to be trapped; drains, privies, and cess-pools to be kept in good order, and subject to the inspection of commissioners. The latter to cause streets to be cleansed, and dirt and ashes removed from houses; refuse to be vested in them. If the dung or soil of any stable, cowhouse, pigstie, &c. be allowed to accumulate for more than fourteen days, notice to be given for its removal within twenty-four hours; and if not removed the dung to be vested in the commissioners, and removed and sold by them. If any person shall allow any dung or filth to accumulate in his house for three days, after the scavenger has applied to remove it, he shall be subject to a fine of not more than forty shillings. If a certificate, signed by a medical officer of health, or by any two legally qualified practitioners, set forth any accumulation as injurious, notice for its removal within twenty-four hours is to be given by the commissioners, on failure of which it becomes their property, and is to be removed and sold by them. Similar provisions are directed against the removal of offensive matter during the day, and against stagnant pools, and other annoyances. Clause 174 provides for the erection of public conveniences. Clause 175 provides for the appointment of a medical officer of health, *which,* we observe, *is not compulsory.* We subjoin this clause and the two following, entire.

"And whereas, the health of the population, especially of the poorer classes, is frequently injured by the prevalence of epidemical and other disorders, and the virulence and extent of such disorders is frequently due and owing to the existence of local causes which are capable of removal, but which have hitherto frequently escaped detection from them, it is expedient that power should be given to appoint a duly qualified medical practitioner for that purpose; Be it therefore enacted, that it shall be lawful for the said commissioners to appoint, subject to the approval of one of her Majesty's principal secretaries of state, a legally-qualified medical practitioner, of skill and experience, to inspect and report periodically on the sanatory condition of any town or district, to ascertain the existence of diseases, more especially epidemics increasing the rates of mortality, and to point out the existence of any nuisances or other local causes which are likely to originate and maintain such diseases and injuriously affect the health of the inhabitants of such town or district, and to take cognizance of the fact of the existence of any contagious disease, and to point out the most efficacious modes for checking or preventing the spread of such diseases, and also to point out the most efficient means for the ventilation of churches, chapels, schools, registered lodging-houses, and other public edifices within the said town or district, and to perform any other duties of a like nature which may be required of him; and such person shall be called the medical officer of health for the town or district for which he shall be appointed; and it shall be lawful for the said commissioners to pay to such officer such salary as shall be approved of by one of her Majesty's principal secretaries of state.

"And be it enacted, that whenever it shall be lawful for any coroner to summon medical witnesses, and to direct the performance of a post-mortem examination, under the provisions of an act passed in the session of Parliament held in the sixth and seventh year of the reign of his late Majesty King William the Fourth, intituled 'An Act to provide for the Attendance and Remuneration of Medical Witnesses at Coroners' Inquests,' it shall be lawful for such coroner to issue his order for the attendance of the medical officer of health for the town or district within which any such inquest shall be held, and to direct the performance by such medical officer of a post-mortem examination, with or without analysis of the contents of the stomach or intestines, without fee or reward; and any provisions contained in the said Act for imposing any penalty on any medical practitioner for any disobedience of any order of such coroner shall be taken to extend and apply to such officer of health.

"And be it enacted, that when it shall appear to the said commissioners, either from the report of the officer of health or otherwise, that any house, or the premises adjoining to any house, is or are in such a filthy or unwholesome condition, that the health of the inmates or of the public is thereby affected or endangered, or that the whitewashing, cleansing, or purifying of any house or part thereof, would tend to prevent or check infectious or contagious disease which may have occurred therein, it shall be lawful for the said commissioners from time to time, if they shall think it expedient, to order the owner or occupier of any house or dwelling, or part thereof, within any town or district, to whitewash, cleanse, and purify the same in such manner and within such time as the said commissioners may deem reasonable; and if such owner or occupier shall not comply with such order, he shall forfeit and pay any sum not exceeding *ten shillings* for every day's neglect thereof; and it shall be lawful for the said commissioners to cause such house or dwelling, or any part thereof, to be whitewashed, cleansed, and purified, and to recover the expense thereof from such owner or occupier; provided that when, on account of the poverty of such owner or occupier, or other special circumstances, it shall appear expedient to the commissioners to pay the whole or any part of such expense, it shall be lawful for them so to do."

Clause 184 is also so important that we give it at length too:

"And be it enacted, that it shall be lawful for the said commissioners, and they are hereby required from time to time to make by-laws as they shall think fit, for all or any of the purposes following; (that is to say,)

"For preventing nuisances and annoyances in any streets, or near thereto, and for effecting cleanliness therein.

"For making regulations for registering and inspection of slaughter-houses and knackers' yards, and for keeping the same in a cleanly and proper state, and for removing filth therefrom at least once in every twenty-four hours, and for requiring that they shall be provided with a sufficient supply of water.

"For regulating the manner of keeping swine, and for preventing the keeping thereof in any dwelling-house, and for describing the limits in such town or district within which it shall be lawful to keep the same.

"For the punishment of persons selling unwholesome meat, and for seizing and condemning the same.

"For regulating the duties of scavengers, and for regulating the management of public privies.

"For making regulations for the registering of lodging-houses, and for maintaining cleanliness therein, and keeping them in a wholesome condition.

"For laying down rules for cleansing filthy and unwholesome dwellings, and to ascertain and fix what pecuniary penalties shall be incurred by persons breaking such laws.

"Provided always, that no such last-mentioned penalty shall exceed for any such offence the sum of five pounds, and in the case of a continuing nuisance the sum of ten shillings for every day during which such nuisance shall be continued or unremedied."

These by-laws are to be approved by the secretary of state, and published in the newspapers.

The supply of water and the extinction of fire are provided for in clauses 190 to 251. The power granted to the commissioners for these purposes are of the most extensive description; they constitute them, in fact, a little civic parliament, possessed of more authority than corporations or other existing public bodies.

Clause 250 merits notice, as it provides for the establishment, by the commissioners, of a "humane apparatus, with all such matters and things as may appear to them necessary, to assist in searching for drowned persons, and restoring animation to persons apparently drowned, and to employ and reward assistants therein, in such manner as the said commissioners shall deem advisable."

The remaining clauses of the bill are of a financial character, and need no special notice, except that, by clause 254, commissioners are authorized to lay a water rate.

We can scarcely hope or wish that this bill will or may give satisfaction to the profession. The science of medicine holds apparently a very subordinate position in the opinion of its concoctors. The secondary appointment of the officers of health is not even imperative on the local authorities, as it undoubtedly ought to be; while there is no provision made for those higher applications of medical science to political economy, the practicability and propriety of which we have from time to time demonstrated.

III. Appendix to the Report. This, like the appendix to the first report, contains an immense amount of information on matters relating to public hygiene. This information is principally comprised in the local reports of those members of the commission who were itinerant. It is extremely difficult to give a condensed analysis of these reports. In the main features of sewerage, cleansing, and structural arrangements, the towns of England closely resemble each other, and consequently the facts related by each commissioner do not widely differ. In some particulars, however, the towns do differ widely. The manufacturing have few public places of recreation; Birmingham, for example, has no public walk, and there is no place in or near it where the working classes can bathe. The same remark applies to Stourbridge. Mr. Slaney made a discovery here:

"In the immediate vicinity of this neat and flourishing town is the large, straggling, and populous hamlet of King's Swinford, called by the appropriate name of 'Lye a waste.' These waste people are almost all nailers; their houses, or rather huts, are of all forms, grouped in two, three, or more together, over a wide space. Filthy open dithecs, heaps of rubbish and dirt, surround their neglected habitations; disorder and poverty appear on all sides; there are no regulations or attempts at improvement." (p. 209.)

The following observation, by the same gentleman, has a general application, and ought to be seriously considered. Referring to Droitwich, in Worcestershire, Wellington, in Shropshire, and other small towns, he remarks:

"It is matter of remark also, that in many of these places a colony of poor Irish have planted themselves, who are *fast increasing* in numbers: their habitations are habitually dirty and neglected; the frequent source of disease; and the inhabitants are generally, from their frequent quarrels, and the bad example they afford to others, a great trouble to the vicinity." (p. 219.)

The following is Mr. Slaney's summary of the evils arising from the want of proper sanatory regulation:

"1st. Shortening the duration of the lives of the community.

"2d. Disease, suffering, and inability to work on the part of many who survive; the causes of great cost to the country.

"3d. Crime, theft, and the loss of property, which the police constantly point out as arising from these neglected classes.

"4th. Riots, disturbances, and drunkenness, which may generally be traced to the same class of persons, often to the same places.

"5th. Great injury to the education of the poor, which is constantly neutralized in its good effects by the neglect and evils they see around them. The same observation applies to the inestimable advantages of religion, and attendance on religious worship.

"6th. Great discontent in some, and sluggish apathy in others, producing recklessness of conduct, indifference, and want of attachment to the institutions of the country.

"7th. The loss to the humbler classes of the cheapest, best, and most enduring pleasures, viz. those arising from the kindly influence of the domestic relations between husbands and wives, parents and children, brothers and sisters,—that pure source of happiness derived from mutual kindness, attachment, and good offices, is, amid the hardening and disgusting scenes described, almost destroyed." (p. 223, seq.)

Mr. Slaney estimates the pecuniary loss consequent upon these evils: the following is his bill for a town with a population of 20,000:

| | £ |
|---|---|
| "Loss of the labour of those prematurely cut off, as before stated, per annum . . . . . . . | 6500 |
| Loss during illness of those who recover . . . . | 1000 |
| Their support in illness . . . . . . . | 1000 |
| Cost of crime and vice arising from the same causes stated . | 6250 |
| | £14,750" |

The report of Mr. Slaney is followed by that of Sir H. T. De La Beche on Bristol, Bath, and other towns in the West of England. It is illustrated by a geological section and map of Bristol, showing the common seats of fever and cholera, and by a geological map of Bath.

Dr. Lyon Playfair assisted in the report for Bristol. A peculiarity of Bristol is in the existence of parochial conduits—one or two of ancient foundation; there are also many public wells. It appears, however, that there are few towns so inadequately supplied with water. In other respects the sanatory condition of Bristol is low; the buildings and drains badly constructed, slaughter-houses and dead-yards scattered through the town. Indeed, Bristol, with a climate known to be mild and salubrious, enjoys the unenviable celebrity of being the third most unhealthy town in England. The sanatory condition and arrangements of Bath present a favorable contrast to those of Bristol.

Mr. Payne, surgeon, of Frome, supplied Sir H. De La Beche with a report on the health of that town, from which we extract the following statement, as interesting to statisticians.

"There is one great evil which I see adverted to in the interesting report furnished by Mr. Chadwick, and which I know prevails to a great extent as well in this as in other manufacturing towns, and which I consider to be of serious importance, viz. the calling children still-born who have been born alive, but from neglect or intentional ill-usage have soon ceased to exist. This is one great source of the incorrectness of tables relating to statistical investigations. I do not see how these can be correct, when registration takes no cognizance of still-born children, who are, undoubtedly, as much a part of the population, although from neglect or some difficulty in delivery, probably mechanical compression, are to all appearances dead, as others, who, not being exposed to such casualties, are born alive. There can be no question that gross neglect is practised by the loose women who act as midwives to the poor, which adds greatly to the amount of mortality amongst infants, and the consequent evil (generally overlooked) of a quick succession of unhealthy and rickety children. I have frequently seen newly-born infants put aside as still-born that have been lustily formed, and who, in the space of half-an-hour, have astonished the bystanders by loud cries; and others, who, with the ordinary attention and application of the usual remedies generally resorted to on such occasions, have been speedily resuscitated. Indeed, so ignorant and unfeeling are the majority of the poor women of this place, that they would rather see their infants perish than have recourse to any measure, and even censure the adoption of means calculated to recover such as are still-born. I have very frequently met with such myself, and I can assert that it is the common practice; and, from what I have witnessed repeatedly, I can fully corroborate the printed evidence given by other registrars, as reported in Mr. Chadwick's investigations." (p. 293, seq.)

This is really an appalling statement.

Mr. Payne being both union officer and registrar has had opportunities for a careful collection of facts. These he has arranged, and states as the result that "in Frome, 1 in 5·5 of the total deaths was from phthisis, and 1 in 21·8 from pneumonia; 1 in 10·7 was from continued fever and typhus; 1 in 11·8 from debility, chiefly new-born infants; 1 in 10 from diseases of the brain and nervous system; 1 in 23·4 of dropsy; 1 in 49·3 of accidents; and 1 in 5·5 of age."

The town of Swansea is remarkable for copper works, which impregnate the air with sulphureous, sulphuric, and arsenious acids. In consequence of these gaseous products, vegetation is destroyed around the works, particularly westward, towards which the prevalent winds more frequently drive the "copper-smoke," so that on the exposed side of Cilfay Hill no plant can grow, and the very soil is washed from the subjacent gravel and rock, from the absence of protecting vegetation, and where, moreover, the glass in the windows of the town is corroded from the same causes. It becomes an especial object of interest to see how far the condition of the atmosphere may affect the health of the organizations within its influence. Many plants cannot be grown within the range of these vapours even where they become, as it were, diluted with pure air: the colour of the *convolvulus major* has been known to be changed to red, after a few hours' driving of the "copper-smoke," at the distance of two miles from the works; and the horses and cattle that feed upon the grass, where it can grow within the range of much of this smoke, are affected with a great thickening of the knee-joints, and their teeth suffer, so that they must be frequently removed from such localities to preserve them.

Although it might be considered that vapours which clearly caused such destruction to vegetation could scarcely fail to injure the health of persons coming within their range, the general impression seems to be, that these vapours by no means produce the serious consequences to health that might be supposed. It is stated, that in proportion as the copper-works have been erected, ague, which once prevailed in the low grounds near the course of the river, to the northward of the town, has disappeared, so as now to be little known; and it is thought that the copper-smoke greatly counteracts the injurious effects to health which would otherwise arise from the neglected sewerage, drainage, and scavenging of the town.

*The report on the sanatory condition of the large towns in Lancashire* is, we think, the most important document of the kind we have ever perused. It is divided into two parts: the first discussing the civic structural arrangements of the towns situate in the manufacturing emporium of the world; the second determining the results of those arrangements on the health and happiness of the people. In the first part, we have a repetition of the odious and abhorrent details noted before, indicating the same want of hygienic regulations as is observed in other towns. Stagnant pools, unsewered streets, dead dogs and cats, filth of all kinds, cottages stinking like privies,—all usque ad nauseum. But here is something of a novelty in the reeking monotonous gurgle. It is the statement given by a shopkeeper of Manchester, whose rooms, in which he had resided some years, overlook a slaughter-house for pigs:

"What occupation does your neighbour pursue?—He kills pigs, which he gets over from Ireland. Often the pigs, in coming over in the packet, die, and I have seen as many as thirty dead pigs at a time brought into the yard. They are thrown under that shed there, until there is time to cut them up, and by that time I have seen the maggots fairly dropping out of them. Then they are cut up, and I believe are made into salt bacon or sold for sausages. The entrails of such pigs are generally too far gone to be of use, and they are thrown into the dunghill. When the dunghill is stirred up to be taken away, oh! sir, the smell is awful: we are forced to shut our windows and doors, and stuff pieces of cloth into the keyholes; but all this does not keep it out. The entrails of the live pigs killed in the yard are boiled and sold, and give out a very bad smell, but nothing like the others.

"Have you not complained of this nuisance?—Yes, we have; but we were told it was no use complaining, for doctors agreed that these smells were very healthy. (!) Besides, the owner of the yard is a very good neighbour, and tries to keep things as clean as he can, but his occupation beats him in that.

"Is that your only child?—Yes; but it is a poor sickly thing for 15 months old. I thought at one time these smells might have something to do with its being so poorly, but that can't be if they are healthy." (p. 374.)

Who will blame an abstemious Hebrew after reading this?

The details regarding the lodging-houses in Manchester and Liverpool, already referred to, although presenting another aspect, have the same distressing character. Dr. Playfair says,

"I will not dwell upon scenes which I myself have witnessed on entering these dens during the night; but their nature may be easily conceived, and their immoral tendency rendered obvious, when it is considered that the lowest mendicants, thieves, and prostitutes, make these houses their usual abode; and with these abandoned persons the travelling artisan and his family are thus brought into close contact."

The results of these and the other hurtful agents alluded to, are dwelt on at length in the second part. In particular, an elaborate table is given of the pecuniary damages inflicted by them. Mr. Chadwick was the first to show that an excessive mortality from disease and hunger does not retard, but rather accelerates the increase of the population. The proportion of remarriages, Dr. Playfair observes, affords a capital test of the truth of this view, for it is obvious that the death of one of a married couple must take place at an early age, if the relict again marries. The two extremes in Lancashire form powerful proofs of this position; the proportion of remarriages to 100 marriages being only 5·52 in Ulverstone, the most healthy district, and as much as 14·27 in Liverpool, the most unhealthy district. In the ten towns of Lancashire in which the average age at death of adults is the highest, the proportion of remarriages to 100 marriages is 10·41; and in the eleven towns in which the average age at death of adults is the lowest, the proportion is as great as 12·99.

As the premature loss of one member of a family enables the relic to remarry, in this point of view, the centesimal proportion of remarriages becomes a natural index of premature adult mortality. The early death of a male parent in the class of operatives, occasions, in almost every instance, a pecuniary burden upon the surviving relatives, or upon the public at large, part only of which is exhibited by the large amount of widowhood and orphanage dependent on the poor-rates. The table we have referred to gives the general result, without taking into consideration the diminution of the physical and mental energies of the survivors from sickness, and other depressing causes; without estimating the loss from the substitution of young and inexperienced labour for that which is skilful and productive; without including the heavy burdens incident to the large amount of preventible widowhood and orphanage; without calculating the loss from the excess of births, resulting from the excess of deaths, or the cost of the maintenance of an infantile population, nearly one half of which is swept off before it attains two years of age, and about fifty-nine per cent. of which never become adult productive labourers; and with data in every case much below the truth,—Dr. Playfair estimates the actual pecuniary burdens borne by the community in the support of removable disease and death in Lancashire alone, at the annual sum of five millions pounds sterling!

Opiates are extensively used by the lower classes for the purpose of dosing their children, not only in the form of Dalby's carminative and other quack medicines, but in that of pure laudanum. There is an estimate that one druggist supplies 700 families weekly with the "medicine." Only allowing one ounce to each family, Dr. Playfair observes, after some remarks of this kind,

"Thus we have *three* druggists, all of acknowledged respectability (!) in one district of Manchester, selling respectively five and a half, three and a half, and one—in all, nine gallons weekly, two of them testifying that 'almost all the families' of the poor in that district habitually drug their children with opiates; and the third, after a lengthened examination of all the customers who attended a pawnbroker's shop, kept by a relation of his own, giving as a statistical result, that five out of six families in his district were in the habitual use of narcotics for children." (p. 455.)

In Rochdale and other towns, similar evidence is given. "A. B. is in disgust at the assertion that the practice prevails to a *great extent* in Rochdale, stating as the result of his inquiries, that out of ten families of the operatives, *not more than six* are in the habitual use of opiates;" while another druggist, who also had abundant opportunities of knowing the custom, considers "one third of the working people used these sleeping-stuffs." At Clitheroe, 4,000 poppy heads are annually sold for making "sleeping tea" for children. In Rochdale, the poor, finding Godfrey and Dalby (society will heavily curse these men some day) too expensive, have got into the practice of buying at a time a pennyworth of solid opium, and a pennyworth each of anise and carraway seeds; these they boil with sugar and treacle, and dose the children with the mixture. And so Dr. Playfair goes on with fact after fact—all alike, yet all different; dwelling upon them with a praiseworthy but sickening minuteness. The little victims, with a wonderful precocity, are described as stretching out their tiny hands for the bottle, " for they know it, and when they get it, drink it as eagerly as a drunkard empties his glass !" Equally deplorable is the description given by a druggist, who says, " I have seen the little children in the shop put the neck of the bottle in their mouths and bite the cork, so fond are they of the preparation; for coming to the shop so often, they know the bottle!"

Dr. Playfair found it difficult to write as calmly as became a commissioner upon such facts as these.

Our fast diminishing limits warn us, however, to hold our hand; and leaving some important statistical details, as to the hygiene of morals given by Dr. Playfair, pass on to the second volume. This comprises reports on the condition of the towns in the north and midland counties of England, by Dr. Reid, Professor Owen, Mr. J. R. Martin, and Mr. Smith of Deanston. There is also an elaborate report on the condition of the city of Exeter, by Dr. Shapter. The notabilia in this volume are, firstly, that Dr. Reid in his report on the northern coal-mine district, sets forth the principles of ventilation as applicable to dwellings, mines, factories, churches, schools, &c.; illustrating his views by twenty-one coloured lithographs, containing nearly one hundred figures! Dr. Reid shows also the intimate connexion between cholera, typhus, and the ordinary sources of malarious emanations.

Mr. Martin, in his report on the state of Nottingham, Coventry, &c., sets forth certain questions which he addressed to Sir James Macgregor, as to the condition of the recruits obtained from the manufacturing districts. The answers are striking, and corroborate similar observations made in France. (Vide our Fourteenth Volume, p. 457.) Sir James observes,

" It is a matter of every day experience that the physical and moral qualities of the country recruits are of a much higher order than are met with in men who have been reared in large towns. In the former, the bodily and constitutional powers are in general much greater than in the latter. There is much less proneness to disease in countrymen, while the power of withstanding the inroads of sickness is in them considerably greater, and they are far more capable of enduring the fatigues and privations to which soldiers may be subjected than recruits of the latter class, in whom the stamina at best have been but imperfectly developed, and whose general health has been depreciated by the nature of their occupations and habits,—impure air, want of proper exercise, and the many debilitating and morbific influences inseparably connected with the condition of artisans in large towns.

" 2d. For these and similar reasons, there exists a marked superiority, both physically and morally, in the capabilities for foreign service of the country recruits over those possessed by townsmen." (vol. ii, p. 131.)

About one fifth only of the country recruits are medically rejected, while nearly one half of the civic are found unfit.

Mr. Martin notices the one-eyedness of honest folk, when giving a sanatory character of their own locality.

" The observations of lay persons on questions affecting public health are of great importance; indeed, the experience of observant laymen has not been sufficiently regarded by professional inquirers. But, admitting its importance, there are yet some circumstances connected with lay evidence requiring that we should sometimes receive it with reserve. One of these drawbacks consists in the impression so common amongst citizens, that *their* city or town forms an exception to the rule of unhealthiness; indeed, they receive any intimation to the contrary as an impeachment (*stigma* is the term they use,) of the character, moral as well as physical, of their favorite locality; and so strong is this feeling, that men of the highest integrity and intelligence will be found continually to deceive themselves, and, unintentionally, they will endeavour to deceive others on such points. . . . . . But laxity and want of accurate observation is not exclusively confined to the lay community; for sometimes we have an exordium of the following character from quarters whence a more exact information might be expected: 'This town, I consider, upon the whole, one of the healthiest towns in the kingdom.' Again, of another town (like the last), most unhealthy, we have it reported, that 'the general condition of the town is comparatively healthy: at the present time it may be considered in a very healthy state.' It is but justice to state, however, that, in general, throughout the entire extent of my personal examination, I found the medical witnesses alike intelligent and humane." (vol. ii, p. 129, seq.)

Mr. James Smith notices another "element" of hinderance; it is singular that he alone makes the observation.

"There is another element which presented itself as a serious barrier to the carrying out of local works by the authorities as at present constituted—namely, party divisions. At Hull, when I visited the town, and paid my respects to the mayor as a public officer, without knowing or thinking it my duty to notice of what political party he was, and made inquiries of the municipal officers, and perambulated the poorer districts with, I found that this very innocent act of mine was regarded, by very respectable persons, but an anti corporation party, as 'taking a side.'" (Ibid. p 163.)

The most remarkable portion of Mr. Smith's report is his essay on the application of sewer water to agricultural purposes. He proposes to irrigate the arable land, around our large towns with it, as a substitute for guano, using pipes and steam power for distributing it. In making an estimate of the cost, Mr. Smith observes:

"I have confined the district to be supplied to an area of four square miles, containing 2560 statute acres I have supposed the whole to be laid off in 10-acre fields, and have put down the position for the service-pipes in such order, as to effect the distribution of the water over each area of 40 acres by a hose-pipe, 312 yards long The main piping I have assumed at the length of the side of the square, with one mile added to clear the suburbs of the town. The main is taken at 12 inches in diameter, which will be sufficient to pass the quantity of water required for a great extent of land; and the service pipes are taken at 4 inches diameter, which is very ample, as never more than two or three jets will be playing from one service pipe at the same time. The main pipes I have estimated as of cast-iron, the service pipes as of fire-clay, as I have ascertained that such can be had at one third of the price of cast iron pipes, and I have seen such proved to a pressure of 600 feet. They will certainly stand a pressure of 300 feet. These pipes I suppose to be sunk two feet under the surface, with a plug opening for attaching the hose for each four fields or 40 acres. The hose pipe and jet must in all cases be worked by persons employed by the sewer-water establishment, who will apply the liquid at such times, in such manner, and in such quantity as the farmer shall desire, under proper regulations. Part may be delivered by jet, part for purposes of irrigation; and it is evident that any farmer would be greatly benefited by appropriating a portion of his farm as meadow, to be irrigated by the sewer-water for the production of early and abundant crops of grass." (Ibid. p 176.)

From various data he calculates, that the cost of manuring one acre with sewer-water, in strength equal to 2½ cwt. with guano, costing 1*l*., or to fifteen tons of farm-yard manure costing 3*l*., will amount only to 12*s*. 9*d*. The net income from the sewer-water of a town is estimated at nearly 1*l*. per head.

"Taking a general view of the subject, we may safely assume a clear revenue from the sewer-water of all towns of 1*l*. for each inhabitant, either in a direct money return, or partly to the inhabitants in a reduced price from the increased abundance of produce; and it is obvious, that such income, annually accruing, will provide a sufficient fund for the improvement of all towns, in a manner corresponding to the most enlightened views with respect to sanatory regulation and improvement of the present time, and will remain as a source for accomplishing such further improvements as science and practical experience shall from time to time suggest." (p. 478.)

An insuperable bar to the accomplishment of Mr. Smith's views will be found, we suspect, in bucolic stupidity.

We here terminate our notice of the labours of the health of towns commission. The four volumes present an invaluable repository of facts bearing on public hygiene, collected at a great expense, by most competent observers, from some of the largest and most important towns and cities in the world. As books of reference they are essential to the practitioner's library; none should be without them; and no practitioner should neglect their perusal, because, sooner or later he will certainly be called upon to act in his professional capacity, in respect of one or other of the numerous matters and things bearing on the public hygiene of his country.

---

